# Joint Resource Management and Trajectory Optimization for UAV-Enabled Maritime Network

**DOI:** 10.3390/s22249763

**Published:** 2022-12-13

**Authors:** Guanding Yu, Xin Ding, Shengli Liu

**Affiliations:** College of Information Science and Electronic Engineering, Zhejiang University, Hangzhou 310027, China

**Keywords:** maritime communication network, UAV, resource management, trajectory design

## Abstract

Due to the lack of places to employ communication infrastructures, there are many coverage blind zones in maritime communication networks. Benefiting from the high flexibility and maneuverability, unmanned aerial vehicles (UAVs) have been proposed as a promising method to provide broadband maritime coverage for these blind zones. In this paper, a multi-UAV-enabled maritime communication model is proposed, where UAVs are deployed to provide the transmission service for maritime users. To improve the performance of the maritime communication systems, an optimization problem is formulated to maximize the minimum average throughput among all users by jointly optimizing the user association, power allocation, and UAV trajectory. To derive the solutions with a low computational complexity, we decompose this problem into three subproblems, namely user association optimization, power allocation optimization, and UAV trajectory optimization. Then, a joint iterative algorithm is developed to achieve the solutions based on the successive convex approximation and interior-point methods. Extensive simulation results validate the effectiveness of the proposed algorithm and demonstrate that UAVs can be used to enhance the maritime coverage.

## 1. Introduction

### 1.1. Background

Maritime communication plays an important role in many maritime applications including oil exploitation, disaster warning, and military surveillance. All these scenarios require higher data rate and more reliable wireless communication. However, these requirements cannot be met by the existing maritime communication networks, which only reach a few Mbps [1,2]. At present, without terrestrial cellular network, very-high-frequency (VHF) or satellite technologies are adopted for maritime communication [3]. While the satellite can provide wide coverage, it suffers from some problems, such as large communication delay and expensive charge. On the other hand, the on-shore base stations (BSs) can only provide a limited offshore coverage, where the coverage blind zones are inevitable. These mean that the existing infrastructures cannot meet the increasing maritime communication demands.

To overcome the problems of traditional maritime wireless communication, unmanned air vehicles (UAVs) have been proposed as a promising method to provide high-rate and stable communications for maritime users with a weak channel state [4,5,6]. Benefiting from their agile mobility, UAVs have a larger coverage range than terrestrial base stations (TBSs) and they are more appropriate for mobile users when deployed as aerial mobile base stations [7,8]. In addition, UAVs are cost-effective for large-scale applications in a maritime scenario. However, current studies mainly focus on UAV communication in the terrestrial scenario [9]. Due to the unique environment on the ocean, there are many challenges to apply UAVs in maritime wireless communication.

### 1.2. Related Works

There have been several studies on the maritime channel model and the transmission efficiency of on-shore BSs. The authors of [10] made a detailed investigation on the channel model of the air-to-sea and near-sea-surface links. It is claimed that the maritime channels are different from the conventional terrestrial wireless channels due to the complex maritime environment such as sparse scattering, sea wave movement, and the ducting effect over the sea surface. In [11], the authors declared that the two-ray signal transmission model with the line-of-sight (LoS) ray and the sea-reflected ray is more appropriate than other models for maritime communication. The authors in [12] estimated the maritime users’ channel state information (CSI) by utilizing the shipping lane information of users. Accordingly, a resource allocation scheme was proposed to enlarge the coverage range. To exploit the feature that location-dependent large-scale fading dominates the channel condition, the authors of [13] designed a hybrid preceding scheme for on-shore BSs. To reduce the complexity of this system, only the large-scale channel state information and transmitter was considered. In addition, a long-term evolution was introduced in [14] for the LTE-maritime in South Korea to develop on-shore infrastructures supporting data transmission within the communication coverage of 100 km.

In addition to deploying on-shore BSs, unmanned surface vehicles (USVs) and UAVs have also been applied for coverage enhancement. The authors in [3] considered a USV-enabled maritime network and optimized the minimum expected throughput by establishing the systematic USV kinetics and information transmission models. Similarly, the authors in [15] utilized the USV to collect maritime data, where a target-oriented double deep Q-learning network was designed to minimize the energy consumption and data loss. However, the equipment deployed on the ocean may be affected by the sea surface movement, which may lead to frequent link breakages. Differently, some researchers adopted UAVs as aerial BSs to enhance the coverage of the maritime network. In [16], the authors optimized the UAV trajectory and transmission power to serve the ships through combining satellites and on-shore BSs. Moreover, the coexistence issue between UAVs, satellites, and terrestrial BSs was also discussed. In [17], a cognitive mobile computing network was proposed for search and rescue by using UAVs and USVs, where the search path and communication throughput were optimized by reinforcement learning (RL). The authors of [2] designed a UAV trajectory adjustment algorithm based on dual Q-learning. By adjusting UAVs’ flight stereo-attitudes in real time, they improved the network performance. In [6], the authors employed non-orthogonal multiple access (NOMA) to maximize the minimum ship throughput by solving a joint power and transmission duration allocation problem.

In addition, there have been some studies focusing on the terrestrial scenario. For example, the authors of [18] deployed UAVs as BSs, and the 3D trajectories of UAVs were optimized to increase both the total coverage and the coverage lifetime. A UAV-enabled space-air-ground integrated Internet of Things was proposed in [19], where the system rate was maximized by jointly optimizing the trajectory of the UAV and transmission power of BS. To provide reliable aerial communication, the authors of [20] proposed a quality of service provisioning framework for a UAV-assisted aerial ad hoc network. By considering dynamic aerial ad hoc environments, the UAV’s aerial mobility and service parameters were optimized. However, the energy consumption has not been considered in this work. The influence of the limited on-board energy constraint on system performance is also great. In [21], the authors proposed the J-MATE model to jointly optimize the energy and throughput under the battery constraints. By optimizing the circular trajectory of the UAV around its BS, the energy efficiency of the UAV communication was maximized in [22] by considering the energy consumption of UAV propulsion and throughput.

### 1.3. Motivations and Contributions

Generally, the ships on the sea have fixed shipping lanes [1]. Therefore, with this information, the UAVs are more appropriate for maritime coverage enhancement [23]. However, though maritime UAVs face some similar problems to the terrestrial case, e.g., limited on-board energy and spectrum scarcity problem, they have some unique challenges. First, the hash maritime environment may affect the deployment of UAVs. Different from the terrestrial case, the maritime environment is greatly affected by the weather, such as typhoon and waves. Secondly, since UAVs are parts of the maritime communication networks (MCNs), they rely on the existing MCNs for backhaul links. Regarding the hybrid network architecture, the UAVs need to jointly allocate their communication resources and coordinate interference. Finally, different from previous works that utilize the free space loss model to simplify analysis in terrestrial scenarios [24,25], it is crucial to consider the unique channel model for maritime communication.

To tackle these challenges, in this paper, we consider a multi-UAV enabled maritime network for large-scale maritime communication service, especially, in blind zones. With the practical maritime channel model, the average data rate is maximized by jointly adjusting the user association, power allocation, and UAV trajectory. The main contributions of this paper are summarized as follows.

- A multi-UAV enabled maritime network model is proposed to enhance the coverage of the maritime communication. To improve the maritime performance, the user association, power allocation, and the UAV trajectory are jointly optimized. Specially, the maritime two-ray model, which includes the LoS ray and sea-reflected ray, is adopted to analyze the throughput of the maritime user. Then, an optimization problem is formulated to maximize the average achievable data rate, which is an NP-hard mixed integer nonlinear programming (MINLP) problem.
- To achieve the solutions with a low computational complexity, the optimization problem is decomposed into three subproblems, namely user association optimization, power allocation optimization, and UAV trajectory optimization. Consequently, the successive convex approximation (SCA) technique and block coordinate descent (BCD) method are adopted to solve the corresponding subproblems. Based on this, a joint iterative algorithm is developed.
- We provide some analytical results on the computational complexity and convergence of the proposed algorithm. In addition, extensive simulations are carried out to demonstrate the effectiveness of the proposed algorithm, and that the proposed algorithm converges well and improve the performance of the maritime network. The results also demonstrates that the proposed algorithm exhibits a low computational complexity.

### 1.4. Organization

The rest of this paper is organized as follows. In Section II, we introduce the system model of maritime UAV communication and the problem formulation. Then, an efficient optimization algorithm is proposed to obtain the maximum average rate among all maritime users in Section III. In Section IV, we analyze the convergence and complexity of the proposed algorithm. Experimental results are presented to verify the proposed algorithm in Section V. Finally, Section VI concludes the whole paper.

## 2. System Model and Problem Formulation

In this section, we first describe the system model and then formulate an optimization problem. Table 1 presents the list of symbols used in this paper.

### 2.1. System Model

**Figure 1:**
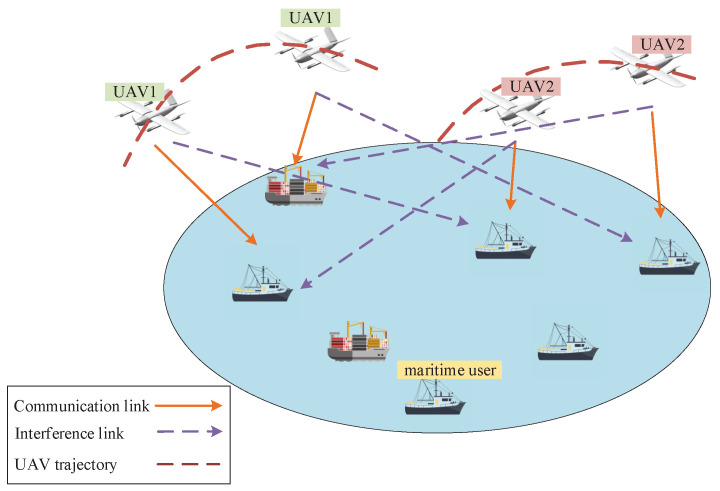
The multi-UAV-enabled maritime wireless network.

As shown in Figure 1, we consider a maritime wireless communication system, where *M* UAVs are deployed to serve *K* users in the open sea. These UAVs and maritime users are denoted by sets M and K, respectively. Regarding the limited spectrum resources, all UAVs reuse the same frequency to provide downlink services for the maritime users in a flight duration of *T*. These users share the downlink frequency with the time division multiple access (TDMA) technique. Considering the hash maritime environment, these maritime UAVs can adopt the design in [26], which can have a better performance than traditional UAVs.

Considering that the moving range of maritime users is relatively small in a flight duration, it is assumed that the user’s position in a flight duration *T* is fixed. The horizontal coordinate of the user *k* can be denoted as $w_{k}=\left(x_{k},y_{k},z_{k}\right)$. To simplify the theoretical analysis, it is assumed that all UAVs connect with users at a fixed height of *H*, which can make UAVs fly smoothly without rising or falling frequently. Hence, the trajectories of UAVs at the time *t* can be denoted by $q_{m}\left(t\right)=\left(x_{m}\left(t\right),y_{m}\left(t\right),H\right)$, where t∈T. It is also assumed that UAVs have all the information of each user, such as CSI, the maritime users locations, and so on. For the convenience of analysis, the flight duration is divided into *N* time slots with the length of $\delta_{t}$. Therefore, the trajectory of UAV *m* can be denoted by $q_{m}\left(n\right)=\left(x_{m}\left(n\right),y_{m}\left(n\right),H\right)$ for n=1,⋯,N. In general, the UAV trajectory satisfies the following constraint

(1) $${∥q_{m}\left[n+1\right]-q_{m}\left[n\right]∥}^{2}\leq d_{\max}^{2},n=1,\cdots ,N-1,\forall m,$$

where $d_{\max}=V_{\max}\delta_{t}$ represents the maximum distance that the UAV can fly in a time slot, $V_{\max}$ indicates the maximum speed of the UAV, and ||·|| is the Euclidean distance. To avoid the collision between UAVs, the following constraints should also be met

(2) $$d_{\min}^{2}\leq {∥q_{m}\left[n\right]-q_{j}\left[n\right]∥}^{2},\forall n,m,j\neq m,$$

where $d_{\min}$ denotes the minimum collision-proof distance. Since the places where UAVs can park on the sea are very limited, we consider that UAVs return to the starting point after a fight duration. Then, they can replenish power to better complete the next flight duration. Hence, the corresponding constraint can be written as

(3) $$q_{m}\left[1\right]=q_{m}\left[N\right],\forall m.$$

Assume that the number of users is larger than the number of UAVs. We adopt a binary variable $a_{k,m}\left[n\right]$ to indicate the connection between user *k* and UAV *m* at time slot *n*. Specifically, when $a_{k,m}\left[n\right]=1$, it means user *k* is connecting with UAV *m*. Otherwise, $a_{k,m}\left[n\right]=0$. Without a loss of system performance, it is assumed that in one time slot, each UAV can only serve one user and each user can only access one UAV at most. Hence, $a_{k,m}\left[n\right]$ satisfies

(4) $$a_{k,m}\left[n\right]\in \left\{0,1\right\},\forall k,m,n,$$

(5) $$\sum_{k=1}^{K}a_{k,m}\left[n\right]\leq 1,\forall m,n,$$

(6) $$\sum_{m=1}^{M}a_{k,m}\left[n\right]\leq 1,\forall k,n.$$

For battery-powered UAVs, the limited energy consists of two parts, i.e., the communication energy and the propulsion energy. Regarding the limited energy of UAVs, we restrict the flight duration and transmission power of UAVs. Besides, we also consider the transmission interference between UAVs, so the restriction on the transmission power can be given by

(7) $$0\leq p_{k,m}\left[n\right]\leq P_{\max},\forall k,m,n,$$

where $P_{\max}$ is the maximum transmission power of the UAV.

### 2.2. Throughput Analysis

The maritime channel models have been comprehensively investigated [10]. According to the existing results [11], the two-ray signal transmission model, including the LoS ray and the sea-reflected ray, is more appropriate for maritime communication. Based on this model, the channel power gain from UAV *m* to user *k* can be expressed as

(8) $$h_{k,m}\left[n\right]={\left[\frac{\lambda }{2\pi d_{k,m}\left[n\right]}\sin\left(\frac{2\pi h_{k}h_{m}}{\lambda d_{k,m}\left[n\right]}\right)\right]}^{2},$$

where λ denotes the carrier frequency wavelength, $h_{k}$ and $h_{m}$ represent the antenna height of user *k* and UAV *m*, respectively, and $d_{k,m}\left[n\right]=\sqrt{{∥q_{m}\left[n\right]-w_{k}∥}^{2}}$ is the distance between user *k* and UAV *m* at time slot *n*.

Since the maritime radio propagations are sparse, the Doppler shift needs to be considered in maritime wireless communication [27]. In this paper, we assume that the Doppler shift can be perfectly compensated by the phase rotation to simplify the analysis [28]. As mentioned earlier, all UAVs use the same frequency band to serve the maritime users. Hence, the co-channel interference between UAVs should be considered. Then, the achievable data rate of user *k* served by UAV *m* can be written as

(9) $$R_{k,m}\left[n\right]=\log_{2}\left(1+\frac{p_{k,m}\left[n\right]h_{k,m}\left[n\right]}{\sum_{j=1,j\neq m}^{M}p_{k,j}\left[n\right]h_{k,j}\left[n\right]+\sigma^{2}}\right),$$

where $\sigma^{2}$ denotes the noise power. Therefore, the average achievable data rate of user *k* during the total flight duration can be expressed as

(10) $$R_{k}=\frac{1}{N}\sum_{n=1}^{N}\sum_{m=1}^{M}a_{k,m}\left[n\right]R_{k,m}\left[n\right].$$

### 2.3. Problem Formulation

For notation simplicity, we denote $A=\{a_{k,m}\left[n\right],\forall k,m,n\}$ as the set of the communication scheduling variables, $P=\{p_{k,m}\left[n\right],\forall k,m,n\}$ as the set of all UAVs’ transmission power variables, and $Q=\{q_{k,m}\left[n\right],\forall k,m,n\}$ as the set of all UAVs’ trajectory variables.

Considering the fairness of the maritime users’ throughput, we aim to maximize the minimum average rate among all maritime users via jointly optimizing the user association, transmission power, and all UAVs’ trajectories. Hence, the problem is formulated as

(11) $$\max_{A,P,Q}\;\min\;R_{k},$$

(12) $$\begin{array}{cc} s.t.\; & \sum_{k=1}^{K}a_{k,m}\left[n\right]\leq 1,\forall m,n, \end{array}$$

(13) $$\begin{array}{cc} \; & \sum_{m=1}^{M}a_{k,m}\left[n\right]\leq 1,\forall k,n, \end{array}$$

(14) $$\begin{array}{cc} \; & a_{k,m}\left[n\right]\in \left\{0,1\right\},\forall k,m,n, \end{array}$$

(15) $$\begin{array}{cc} \; & 0\leq p_{k,m}\left[n\right]\leq P_{\max},\forall k,m,n, \end{array}$$

(16) $$\begin{array}{cc} \; & q_{m}\left[1\right]=q_{m}\left[N\right],\forall m, \end{array}$$

(17) $$\begin{array}{cc} \; & {∥q_{m}\left[n+1\right]-q_{m}\left[n\right]∥}^{2}\leq d_{\max}^{2},n=1,\cdots ,N-1,\forall m, \end{array}$$

(18) $$\begin{array}{cc} \; & d_{\min}^{2}\leq {∥q_{m}\left[n\right]-q_{j}\left[n\right]∥}^{2},\forall n,m,j\neq m. \end{array}$$

Obviously, the problem in (11) is an MINLP problem. It is intractable to directly solve this problem by using conventional convex optimization methods. We will solve this problem in the next section by problem decomposition.

## 3. Joint User Association, Power Allocation, and Trajectory Optimization

In this section, we first decompose the problem in (11) into three subproblems, namely user association optimization, power allocation optimization, and UAV trajectory optimization. Then, the optimal solutions of these three subproblems are developed, respectively.

### 3.1. Problem Decomposition

To obtain the solution, we introduce a slack variable η. Then, an equivalent problem can be given as

(19) $$\max_{A,P,Q,\eta }\;\eta ,$$

(20) $$\begin{array}{cc} s.t.\; & \frac{1}{N}\sum_{n=1}^{N}\sum_{m=1}^{M}a_{k,m}\left[n\right]R_{k,m}\left[n\right]\geq \eta , \end{array}$$

and (12)–(18). Obviously, the above problem is still an MINLP problem and NP-hard [29], since the variables A, P, and Q are jointly coupled in the problem. On the one hand, this problem includes integer constraints, where the variable A is a binary parameter vector. On the other hand, when we fix A and another parameter vector P or Q, this problem is still non-convex and challenging to solve. To obtain the solutions, these variables should be decoupled. Then, the problem in (11) can be decomposed into three subproblems: user association optimization, power allocation optimization, and UAV trajectory optimization.

### 3.2. User Association Optimization

Given the power allocation P and UAV trajectory Q, the user association optimization problem can be first expressed as

(21) $$\max_{A,\eta }\;\eta ,$$

(22) $$\begin{array}{cc} s.t.\;\; & \frac{1}{N}\sum_{n=1}^{N}\sum_{m=1}^{M}a_{k,m}\left[n\right]R_{k,m}\left[n\right]\geq \eta , \end{array}$$

(23) $$\begin{array}{cc} \; & \sum_{k=1}^{K}a_{k,m}\left[n\right]\leq 1,\forall m,n, \end{array}$$

(24) $$\begin{array}{cc} \; & \sum_{m=1}^{M}a_{k,m}\left[n\right]\leq 1,\forall k,n, \end{array}$$

(25) $$\begin{array}{cc} \; & a_{k,m}\left[n\right]\in \left\{0,1\right\},\forall k,m,n. \end{array}$$

It is obvious that this problem is a mixed integer linear programming (MILP) problem and the optimal solution cannot be directly obtained in general. To achieve the feasible solution with a low complexity, the problem in (21) can be transformed by relaxing the binary variable $a_{k,m}\left[n\right]$, as

(26) $$\max_{A,\eta }\;\;\eta ,$$

(27) $$\begin{array}{cc} s.t.\; & 0\leq a_{k,m}\left[n\right]\leq 1,\forall k,m,n, \end{array}$$

(22)–(24).

The above problem in (26) is now convex since the objective problem and all constraints are linear with respect to $a_{k,m}\left[n\right]$ and η. Hence, we can efficiently solve this optimization problem by the interior-point methods [30].

### 3.3. Power Allocation Optimization

Similarly, given user association A and UAV trajectory Q, the problem in (11) can be expressed as

(28) $$\max_{P,\eta }\;\eta ,$$

(29) $$\begin{array}{cc} s.t.\;\; & \frac{1}{N}\sum_{n=1}^{N}\sum_{m=1}^{M}a_{k,m}\left[n\right]R_{k,m}\left[n\right]\geq \eta , \end{array}$$

(30) $$\begin{array}{cc} \; & 0\leq p_{k,m}\left[n\right]\leq P_{\max},\forall k,m,n, \end{array}$$

which is also non-convex since $R_{k,m}\left[n\right]$ in (29) is non-convex with respect to $p_{k,m}\left[n\right]$. To solve this problem, we first rewrite $R_{k,m}\left[n\right]$ as

(31) $$\begin{array}{cc} R_{k,m}\left[n\right] & =\log_{2}\left(1+\frac{p_{k,m}\left[n\right]h_{k,m}\left[n\right]}{\sum_{j=1,j\neq m}^{M}p_{k,j}\left[n\right]h_{k,j}\left[n\right]+\sigma^{2}}\right)\\ \; & =\log_{2}\left(\frac{\sum_{j=1}^{M}p_{k,j}\left[n\right]h_{k,j}\left[n\right]+\sigma^{2}}{\sum_{j=1,j\neq m}^{M}p_{k,j}\left[n\right]h_{k,j}\left[n\right]+\sigma^{2}}\right)\\ \; & ={\bar{R}}_{k,m}\left[n\right]-{\hat{R}}_{k,m}\left[n\right], \end{array}$$

where ${\bar{R}}_{k,m}\left[n\right]=\log_{2}\left(\sum_{j=1}^{M}p_{k,j}\left[n\right]h_{k,j}\left[n\right]+\sigma^{2}\right)$ and ${\hat{R}}_{k,m}\left[n\right]=\log_{2}\left(\sum_{j=1,j\neq m}^{M}p_{k,j}\left[n\right]h_{k,j}\left[n\right]\right.\left.+\sigma^{2}\right)$. However, ${\bar{R}}_{k,m}\left[n\right]$ and ${\hat{R}}_{k,m}\left[n\right]$ both are concave for variable P. Since the result of subtracting two concave functions is not necessarily concave, $R_{k,m}\left[n\right]$ should be further simplified. By using the SCA technique [31,32,33], a convex expression can be derived to approximate ${\hat{R}}_{k,m}\left[n\right]$, which is described in Lemma 1.

**Lemma** **1.** *For any given local point $P^{l}=\left\{p_{k,m}^{l}\left[n\right],\forall k,m,n\right\}$, the following inequality holds*

(32) $$\begin{array}{cc} {\hat{R}}_{k,m}\left[n\right] & =\log_{2}\left(\sum_{j=1,j\neq m}^{M}p_{k,j}\left[n\right]h_{k,j}\left[n\right]+\sigma^{2}\right)\\ \; & \leq \sum_{j=1,j\neq m}^{M}B_{k,j}\left[n\right]\left(p_{k,j}\left[n\right]-p_{k,j}^{l}\left[n\right]\right)\\ \; & +\log_{2}\left(\sum_{j=1,j\neq m}^{M}p_{k,j}^{l}\left[n\right]h_{k,j}\left[n\right]+\sigma^{2}\right)\\ \; & ≜{\hat{R}}_{k,m}^{up}\left[n\right], \end{array}$$

*where $B_{k,j}\left[n\right]$ is the coefficient of the Taylor expansion, and it is given in Appendix A.*

**Proof.**  Please refer to Appendix A.    □

With the above formula transformation, the problem in (28) can be re-formulated as

(33) $$\max_{P,\eta }\;\eta ,$$

(34) $$\begin{array}{cc} s.t.\;\; & \frac{1}{N}\sum_{n=1}^{N}\sum_{m=1}^{M}a_{k,m}\left[n\right]\left({\bar{R}}_{k,m}\left[n\right]-{\hat{R}}_{k,m}^{up}\left[n\right]\right)\geq \eta , \end{array}$$

(35) $$\begin{array}{cc} \; & 0\leq p_{k,m}\left[n\right]\leq P_{\max},\forall k,m,n. \end{array}$$

The constraint (34) is convex with respect to $p_{k,m}\left[n\right]$, and the constraint (35) is a linear constraint with respect to $p_{k,m}\left[n\right]$. As a result, the problem in (33) is a convex optimization problem. Thus, a suboptimal solution of transmission power to the problem in (28) can be efficiently solved by CVX.

### 3.4. UAV Trajectory Optimization

Given user association A and power allocation P, the problem in (11) can be rewritten as

(36) $$\max_{Q,\eta }\;\eta ,$$

(37) $$\begin{array}{cc} s.t.\; & \frac{1}{N}\sum_{n=1}^{N}\sum_{m=1}^{M}a_{k,m}\left[n\right]R_{k,m}\left[n\right]\geq \eta , \end{array}$$

(38) $$\begin{array}{cc} \; & q_{m}\left[1\right]=q_{m}\left[N\right],\forall m, \end{array}$$

(39) $$\begin{array}{cc} \; & {∥q_{m}\left[n+1\right]-q_{m}\left[n\right]∥}^{2}\leq d_{\max}^{2},n=1,\cdots ,N-1,\forall m, \end{array}$$

(40) $$\begin{array}{cc} \; & d_{\min}^{2}\leq {∥q_{m}\left[n\right]-q_{j}\left[n\right]∥}^{2},\forall n,m,j\neq m, \end{array}$$

which is still a non-convex problem due to the throughput-related constraint (37) and the collision avoidance constraint (40). Thus, it is difficult to solve this problem efficiently. To address this challenge, we adopt an approximate method by using the SCA technique for the UAV trajectory optimization problem in (36).

For the constraint (37), the following formula can be first derived by expanding $R_{k,m}$, as

(41) $$\begin{array}{cc} R_{k,m} & =\log_{2}\left(1+\frac{p_{k,m}\left[n\right]h_{k,m}\left[n\right]}{\sum_{j=1,j\neq m}^{M}p_{k,j}\left[n\right]h_{k,j}\left[n\right]+\sigma^{2}}\right)\\ \; & =\log_{2}\left(1+\frac{p_{k,m}\left[n\right]{\left[\frac{\lambda }{2\pi d_{k,m}\left[n\right]}\sin\left(\frac{2\pi h_{k}h_{m}}{\lambda d_{k,m}\left[n\right]}\right)\right]}^{2}}{\sum_{j=1,j\neq m}^{M}p_{k,j}\left[n\right]{\left[\frac{\lambda }{2\pi d_{k,j}\left[n\right]}\sin\left(\frac{2\pi h_{k}h_{m}}{\lambda d_{k,j}\left[n\right]}\right)\right]}^{2}+\sigma^{2}}\right)\\ \; & \approx \log_{2}\left(1+\frac{\frac{p_{k,m}\left[n\right]{\left(h_{k}h_{m}\right)}^{2}}{{\left({∥q_{m}\left[n\right]-w_{k}∥}^{2}\right)}^{2}}}{\sum_{j=1,j\neq m}^{M}\frac{p_{k,j}\left[n\right]{\left(h_{k}h_{m}\right)}^{2}}{{\left({∥q_{j}\left[n\right]-w_{k}∥}^{2}\right)}^{2}}+\sigma^{2}}\right)\\ \; & ={\overset{ˇ}{R}}_{k,m}\left[n\right]-{\tilde{R}}_{k,m}\left[n\right], \end{array}$$

where ${\overset{ˇ}{R}}_{k,m}\left[n\right]=\log_{2}\left(\sum_{j=1}^{M}\frac{p_{k,j}\left[n\right]{\left(h_{k}h_{m}\right)}^{2}}{{\left({∥q_{j}\left[n\right]-w_{k}∥}^{2}\right)}^{2}}+\sigma^{2}\right)$ and ${\tilde{R}}_{k,m}\left[n\right]=\log_{2}\left(\sum_{j=1,j\neq m}^{M}\frac{p_{k,j}\left[n\right]{\left(h_{k}h_{m}\right)}^{2}}{{\left({∥q_{j}\left[n\right]-w_{k}∥}^{2}\right)}^{2}}\right.\left.+\sigma^{2}\right)$.

In the maritime environment, the distance between the transmitter and the receiver (in the order of kilometers or tens of kilometers) is much higher than the antenna height (in the order of meters or tens of meters) [34]. Hence, the equivalent infinitesimal substitution can be used in (41). When *x* tends to zero, *x* and sinx are equivalent. By this theorem, $\sin\left(\frac{2\pi h_{k}h_{m}}{\lambda d_{k,m}\left[n\right]}\right)$ in (41) can be replaced with $\frac{2\pi h_{k}h_{m}}{\lambda d_{k,m}\left[n\right]}$.

With (41), the problem in (36) can be rewritten as

(42) $$\max_{Q,\eta }\;\eta ,$$

(43) $$\begin{array}{cc} s.t.\; & \frac{1}{N}\sum_{n=1}^{N}\sum_{m=1}^{M}a_{k,m}\left[n\right]\left({\overset{ˇ}{R}}_{k,m}\left[n\right]-{\tilde{R}}_{k,m}\left[n\right]\right)\geq \eta , \end{array}$$

and (38)–(40). However, the problem in (42) is still non-convex since the result of subtracting two convex functions is not necessarily a convex function. The key observation is that although ${\overset{ˇ}{R}}_{k,m}\left[n\right]$ and ${\tilde{R}}_{k,m}\left[n\right]$ are non-convex with respect to $q_{m}\left[n\right]$ in (43), they are both convex with respect to ${∥q_{j}\left[n\right]-w_{k}∥}^{2}$. Hence, by introducing auxiliary variables $\left\{X_{k,j}\left[n\right],j\in M,\forall k,n\right\}$, ${\tilde{R}}_{k,m}\left[n\right]$ can be rewritten as

(44) $${\tilde{R}}_{k,m}\left[n\right]=\log_{2}\left(\sum_{j=1,j\neq m}^{M}\frac{p_{k,j}\left[n\right]{\left(h_{k}h_{m}\right)}^{2}}{X_{k,j}^{2}\left[n\right]}+\sigma^{2}\right),$$

where $X_{k,j}\left[n\right]$ satisfies the following constraint

(45) $$X_{k,j}\left[n\right]\leq {∥q_{j}\left[n\right]-w_{k}∥}^{2},j\in M,\forall k,n.$$

It can be found that ${∥q_{j}\left[n\right]-w_{k}∥}^{2}$ is a convex function with respect to $q_{j}\left[n\right]$, which causes the non-convexity of the problem in (42). Hence, we apply the SCA technique to transform the non-convex constraint. By applying the first-order Taylor expansion at the local point $Q^{l}=\left\{q_{m}^{l}\left[n\right],\forall m,n\right\}$ obtained in the *l*-th iteration, we have

(46) $${∥q_{j}\left[n\right]-w_{k}∥}^{2}\geq Q^{lb}\left[n\right]={∥q_{j}^{l}\left[n\right]-w_{k}∥}^{2}+2{\left(q_{j}^{l}\left[n\right]-w_{k}\right)}^{T}\left(q_{j}\left[n\right]-q_{j}^{l}\left[n\right]\right),$$

which is convex since $Q^{lb}\left[n\right]$ is linear with respect to $q_{j}\left[n\right]$.

However, (44) does not conform to the CVX optimization rule since $X_{k,j}\left[n\right]$ is in the denominator of (44). Therefore, CVX cannot be used to solve the problem in (42) directly. Then by Lemmas 2 and 3, the problem in (42) is re-formulated to satisfy the CVX optimization rule.

**Lemma** **2.** *By introducing auxiliary variable $D_{k,j}\left[n\right]$ to substitute for ${\tilde{R}}_{k,m}\left[n\right]$, i.e.,*

(47) $$D_{k,j}\left[n\right]\geq \log_{2}\left(\sum_{j=1,j\neq m}^{M}e^{z_{k,j}\left[n\right]}+\sigma^{2}\right),$$

*with an additional constraint as*

(48) $$\log_{2}\left(p_{k,j}\left[n\right]{\left(h_{k}h_{m}\right)}^{2}\right)-2\log_{2}\left(X_{k,j}\left[n\right]\right)\leq z_{k,j}\left[n\right],j\in M,\forall k,n,$$

*where $z_{k,j}\left[n\right]$ is the auxiliary variable. The new constraint (47) is convex.*

**Proof.**  Please refer to Appendix B.    □

After the above transformation, ${\tilde{R}}_{k,m}\left[n\right]$ is convex with respect to variable $X_{k,j}\left[n\right]$ and conforms to the CVX optimization rule. Next, we will transform ${\overset{ˇ}{R}}_{k,m}\left[n\right]$ into a linear form, as described in Lemma 3.

**Lemma** **3.** *For any given local point $Q^{l}=\left\{q_{m}^{l}\left[n\right],\forall m,n\right\}$, the following inequality holds*

(49) $$\begin{array}{cc} {\overset{ˇ}{R}}_{k,m}\left[n\right] & \geq \log_{2}\left(\sum_{i=1}^{M}\frac{p_{k,i}\left[n\right]{\left(h_{k}h_{m}\right)}^{2}}{{\left({∥q_{i}^{l}\left[n\right]-w_{k}∥}^{2}\right)}^{2}}+\sigma^{2}\right)\\ \; & +\sum_{j=1}^{M}-I_{k,j}^{l}\left[n\right]\left({∥q_{j}\left[n\right]-w_{k}∥}^{2}-{∥q_{j}^{l}\left[n\right]-w_{k}∥}^{2}\right)\\ \; & ={\overset{ˇ}{R}}_{k,m}^{lb}\left[n\right], \end{array}$$

*where $-I_{k,j}^{l}\left[n\right]$ is the coefficient of Taylor expansion and it is given in Appendix C.*

**Proof.**  Please refer to Appendix C.    □

According to the previous transformation, (43) can be re-expressed as

(50) $$\frac{1}{N}\sum_{n=1}^{N}\sum_{m=1}^{M}a_{k,m}\left[n\right]\left({\overset{ˇ}{R}}_{k,m}^{lb}\left[n\right]-D_{k,m}\left[n\right]\right)\geq \eta ,$$

which is a convex constraint. Similarly, we apply the SCA technique to transform the collision avoidance constraint (40) into a convex one, as

(51) $$d_{\min}\leq {∥q_{m}^{l}\left[n\right]-q_{j}^{l}\left[n\right]∥}^{2}+2{\left(q_{m}^{l}\left[n\right]-q_{j}^{l}\left[n\right]\right)}^{T}\left(q_{m}\left[n\right]-q_{j}\left[n\right]-\left(q_{m}^{l}\left[n\right]-q_{j}^{l}\left[n\right]\right)\right).$$

Therefore, after introducing the auxiliary variables $\left\{X_{k,j}\left[n\right],D_{k,j}\left[n\right],z_{k,j}\left[n\right]\right\}$ and transforming the formulas, the problem in (36) can be rewritten as

(52) $$\max_{Q,\eta }\;\eta ,$$

(53) $$\begin{array}{cc} s.t.\; & \frac{1}{N}\sum_{n=1}^{N}\sum_{m=1}^{M}a_{k,m}\left[n\right]\left({\overset{ˇ}{R}}_{k,m}^{lb}\left[n\right]-D_{k,m}\left[n\right]\right)\geq \eta ,\forall k, \end{array}$$

(54) $$\begin{array}{cc} \; & D_{k,j}\left[n\right]\geq \log_{2}\left(\sum_{j=1,j\neq m}^{M}e^{z_{k,j}\left[n\right]}+\sigma^{2}\right),\forall k,j\in M,n, \end{array}$$

(55) $$\begin{array}{cc} \; & \log_{2}\left(p_{k,j}\left[n\right]{\left(h_{k}h_{m}\right)}^{2}\right)-2\log_{2}\left(X_{k,j}\left[n\right]\right)\leq z_{k,j}\left[n\right], \end{array}$$

(56) $$\begin{array}{cc} \; & X_{k,j}\left[n\right]\leq {∥q_{j}^{l}\left[n\right]-w_{k}∥}^{2}+2{\left(q_{j}^{l}\left[n\right]-w_{k}\right)}^{T}\left(q_{j}\left[n\right]-q_{j}^{l}\left[n\right]\right),\forall k,j\in M,n, \end{array}$$

(57) $$\begin{array}{cc} \; & q_{m}\left[1\right]=q_{m}\left[N\right],\forall m, \end{array}$$

(58) $$\begin{array}{cc} \; & {∥q_{m}\left[n+1\right]-q_{m}\left[n\right]∥}^{2}\leq d_{\max}^{2},n=1,\cdots ,N-1,\forall m,\\ \; & d_{\min}^{2}\leq {∥q_{m}^{l}\left[n\right]-q_{j}^{l}\left[n\right]∥}^{2}+2{\left(q_{m}^{l}\left[n\right]-q_{j}^{l}\left[n\right]\right)}^{T}\left(q_{m}\left[n\right]-q_{j}\left[n\right]-\left(q_{m}^{l}\left[n\right]-q_{j}^{l}\left[n\right]\right)\right), \end{array}$$

(59) $$\begin{array}{cc} \; & \forall n,m,j\neq m, \end{array}$$

where the constraints are all linear or convex. The problem in (52) is now convex and can be solved by CVX directly.

## 4. Proposed Algorithm

In this section, an overall algorithm is proposed to solve the three subproblems in (21), (28) and (36). Then, the convergence and computational complexity of this algorithm are analyzed.

### 4.1. Algorithm Development

According to the previous analysis, in this subsection, we propose an efficient algorithm to jointly optimize user association, power allocation, and UAV trajectory. The optimal solutions $\left\{A,P,Q\right\}$ can be achieved from the above three subproblems in (21), (28) and (36), respectively. By alternatively optimizing these three subproblems, the problem in (11) can be solved with a low complexity, which is concluded in Algorithm 1.
**Algorithm 1** Joint user association, power allocation, and UAV trajectory optimization algorithm**Input:** The iteration index l=0, the maximum number of iteration $N_{max}$, and the convergence threshold ε.**Output:** The optimal user association A, power allocation P, UAV trajectory Q, and the expected throughput *R*.1:Initialize the transmission power $P^{l}$ and the UAV trajectory $Q^{l}$ with any positive values.2:Initialize the user association $A^{l}=\left\{0\right\}$.3:**while** $R^{l}-R^{l-1}\geq \varepsilon$ or $l\leq N_{max}$ **do**4:   Obtain the optimal $A^{l+1}$ for given $P^{l}$ and $Q^{l}$ by solving problem (21).5:   Obtain the optimal $P^{l+1}$ for given $A^{l+1}$ and $Q^{l}$ by solving problem (28).6:   Obtain the optimal $Q^{l+1}$ for given $A^{l+1}$ and $P^{l+1}$ by solving problem (36).7:   Set l=l+1.8:**end while**9:Obtain the optimal user association A, power allocation P, UAV trajectory Q, and the expected throughput *R*.

### 4.2. Convergence Analysis

For convenience, we define $A^{l}=\left\{a_{k,m}^{l}\left[n\right],\forall k,m,n\right\}$, $P^{l}=\left\{p_{k,m}^{l}\left[n\right],\forall k,m,n\right\}$, and $Q^{l}=\left\{q_{m}^{l}\left[n\right],\forall m,n\right\}$ in the *l*-th iteration of Algorithm 1. Let $\eta_{p}^{lb}\left(A^{l},P^{l},Q^{l}\right)$ and $\eta_{q}^{lb}\left(A^{l},P^{l},Q^{l}\right)$ be the objective values of the problems in (33) and (52) in the *l*-th iteration, respectively. According to Step 4 of Algorithm 1, we have

(60) $$\eta \left(A^{l},P^{l},Q^{l}\right)\leq \eta \left(A^{l+1},P^{l},Q^{l}\right).$$

For given local $A^{l+1}$, $P^{l}$ and $Q^{l}$ in Step 5 of Algorithm 1, we have

(61) $$\begin{array}{cc} \eta (A^{l+1},P^{l},Q^{l}) & \overset{a}{=}\eta_{p}^{lb}\left(A^{l+1},P^{l},Q^{l}\right)\\ \; & \overset{b}{\leq }\eta_{p}^{lb}\left(A^{l+1},P^{l+1},Q^{l}\right)\\ \; & \overset{c}{\leq }\eta \left(A^{l+1},P^{l+1},Q^{l}\right), \end{array}$$

where (a) holds since the first-order Taylor expansion in (32) is tight at the given local point $P^{l}$, which means that the problem in (33) at $P^{l}$ has the same objective value as that of the problem in (28). (b) holds since the problem in (33) is solved optimally with solution $P^{l+1}$ in step 5 with given $A^{l+1}$ and $Q^{l}$. $\left(c\right)$ holds since the objective value of the problem in (33) is a lower bound of the problem in (28). The inequality (61) indicates that the objective value of the problem in (33) does not decrease after each iteration. Similarly, given local $A^{l+1}$, $P^{l+1}$, and $Q^{l}$ of Algorithm 1, we also have

(62) $$\begin{array}{cc} \eta (A^{l+1},P^{l+1},Q^{l}) & \overset{a}{=}\eta_{p}^{lb}\left(A^{l+1},P^{l+1},Q^{l}\right)\\ \; & \overset{b}{\leq }\eta_{p}^{lb}\left(A^{l+1},P^{l+1},Q^{l+1}\right)\\ \; & \overset{c}{\leq }\eta \left(A^{l+1},P^{l+1},Q^{l+1}\right). \end{array}$$

According to (60)–(62), we have

(63) $$\eta \left(A^{l},P^{l},Q^{l}\right)\leq \eta \left(A^{l+1},P^{l+1},Q^{l+1}\right),$$

which indicates that the objective value of the problem in (19) is non-decreasing after each iteration of Algorithm 1. Since the maximum value of the problem in (19) is a finite value in the feasible domain, Algorithm 1 can converge. This completes the convergence proof of our proposed algorithm.

### 4.3. Computational Complexity Analysis

In Step 4 of Algorithm 1, since the problem in (26) is solved by the interior-point method, its computational complexity is $O\left(L_{1}{\left(KMN\right)}^{3.5}\right)$, where $L_{1}$ denotes the number of iterations required to update the user association and KMN is the number of variables. In Step 5 of Algorithm 1, the computational complexity of solving the problem (33) is $O\left(L_{2}{\left(KMN\right)}^{3.5}\right)$, where $L_{2}$ denotes the number of iterations required to update the power allocation and KMN is the number of variables. Similarly, in Step 6 of Algorithm 1, the computational complexity of the problem (52) is $O\left(L_{3}{\left(MN\right)}^{3.5}\right)$, where $L_{3}$ denotes the number of iterations required to update the UAV trajectory and MN is the number of variables. Hence, the overall computational complexity of Algorithm 1 is $O\left(L\left(L_{1}{\left(KMN\right)}^{3.5}+L_{2}{\left(KMN\right)}^{3.5}+L_{3}{\left(MN\right)}^{3.5}\right)\right)$, where *L* represents the number of iterations.

## 5. Numerical Simulation

In this section, we verify the performance of the proposed algorithm by simulations. In the simulations, there are six maritime users located within a 2×2 km${}^{2}$ area. Two UAVs are placed to provide communications to maritime users. Moreover, the height of the UAV is fixed at 50 m. Other main parameters are listed in Table 2.

### 5.1. Single UAV

**Figure 2:**
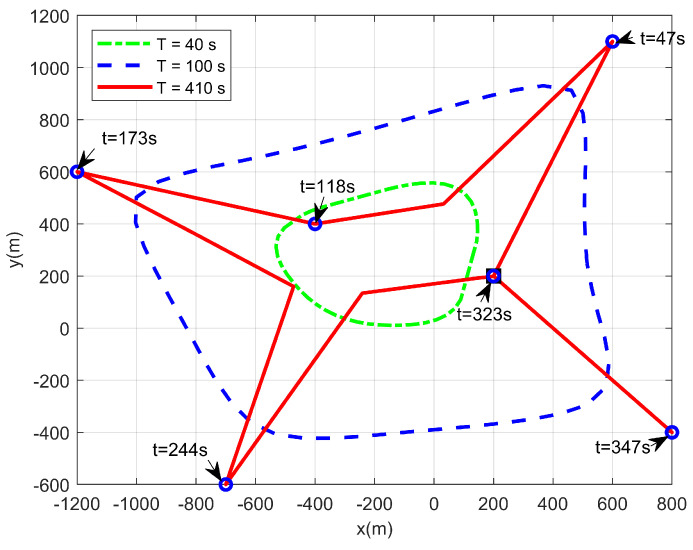
The real−time trajectory of a single UAV.

**Figure 3:**
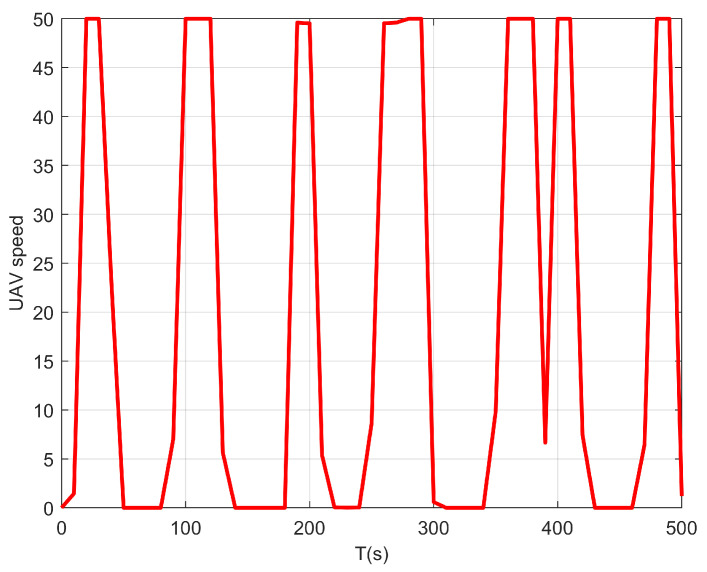
The UAV speed changes within flight duration T=210 s.

We first consider a simple case where only one UAV is taken into consideration, i.e., M=1. Figure 2 shows the real-time horizontal trajectory of a single UAV. Note that the UAV does not need to control the transmission power in this case since there is no interference. Thus, we set $p=P_{\max}$. From this figure, when the flight duration is short, the flight range of the UAV expands as large as possible. This is because the UAV should move closer to the maritime users to obtain a larger achievable rate. With the increase in the flight duration *T*, the coverage range of the UAV becomes larger. When the flight duration *T* is sufficiently large, the flight range of the UAV can cover all maritime users. In this situation, the UAV can approach all users in turn and hover over them for a certain time. Figure 3 presents the speed change of the UAV during the whole flight duration with T=410 s. It can be observed that when the UAV flies close to the maritime user, it will hover over this user. At this time, its speed is close to zero. Then, it will fly to the other users with the maximum speed so that it can provide a better service within a limited flight duration.

**Figure 4:**
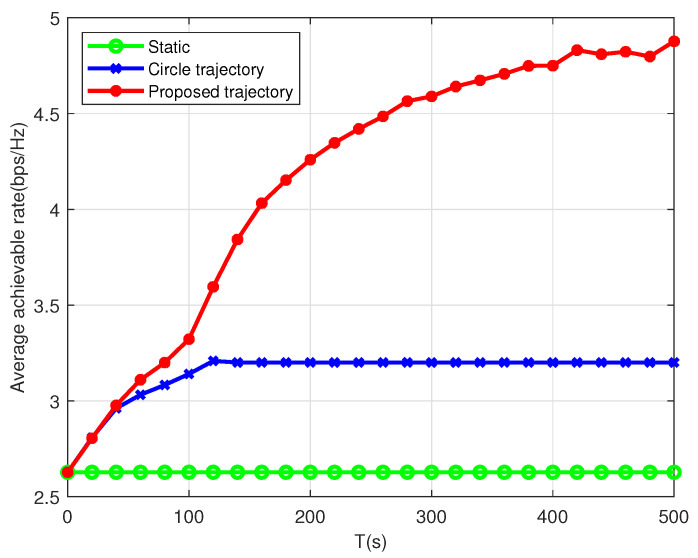
The average achievable rate for the single-UAV scenario versus *T* with different trajectory designs.

As shown in Figure 4, we compare the proposed joint optimization scheme in Algorithm 1 with another two schemes, i.e., static scheme and circle scheme. The static scheme is that the UAV hovers over the middle point of all users, and the circle scheme represents that the UAV flies with a circular trajectory. From this figure, the proposed scheme in Algorithm 1 outperforms the other two schemes. For the static scheme, the average achievable data rate remains unchanged as the flight duration increases, since the distance between the UAV and users is fixed. For the circular scheme, the average achievable data rate first increases and then remains unchanged with the increase in the flight duration *T*. This is because when the flight duration *T* is short, the UAV can only cover a small area and the distance between the UAV and users is long. When *T* is sufficiently large, the UAV can cover all maritime users and provide service for each user. In terms of the proposed algorithm, the average achievable data rate *R* increases as the flight duration *T* increases. When *T* is sufficiently large, *R* increases slowly and finally remains unchanged. This is because when the flight duration *T* is sufficiently large, the UAV has sufficient time to fly close to the maritime users and hovers over them for a certain time. In this situation, the time for flying from one user to another can be negligible and each user can get equal-time service. In this case, the best performance can be achieved compared against the other two schemes.

### 5.2. Multiple UAVs

**Figure 5:**
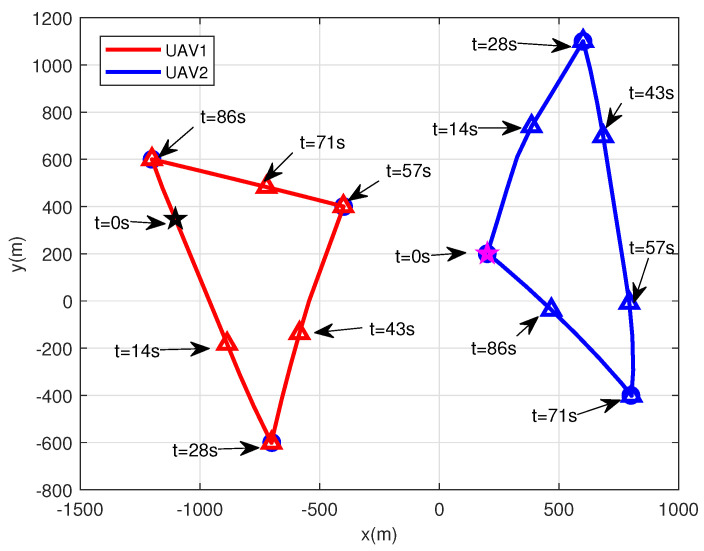
The real−time trajectories of two UAVs for the flight duration T=100 s.

Next, we consider a more general case where two UAVs are deployed to serve multiple maritime users. Figure 5 illustrates the real-time trajectories of the two UAVs within the flight duration T=100 s. As shown in Table 3, instead of hovering over all the users, these two UAVs collaboratively hover over the maritime users close to them and provide service for them. To avoid collision and mutual communication interference, these two UAVs must fly away from each other. However, this strategy will lead to a loss of strong direct communication links when they have to serve two users who are close to each other. By the proposed scheme, since we properly adjust the transmission power of the UAV, both strong direct link and low interference can be achieved, resulting in a larger average achievable data rate.

**Figure 6:**
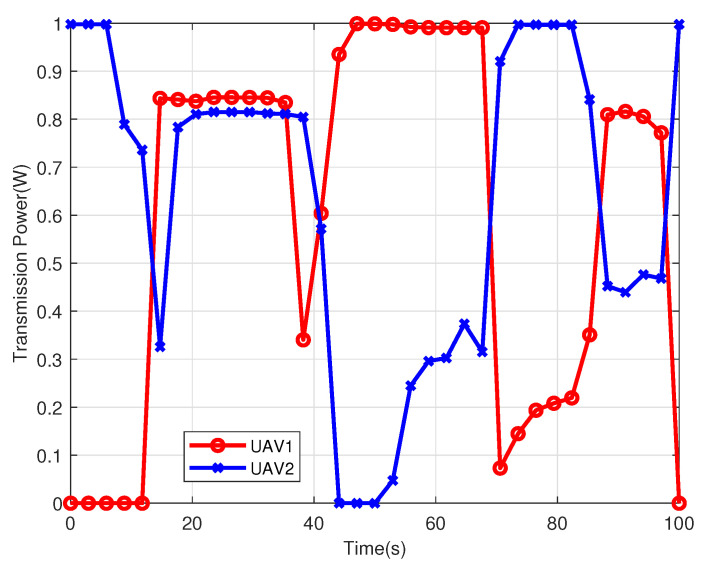
The transmission power changes of two UAVs within the flight duration T=100 s.

Figure 6 illustrates the transmission power of the two UAVs within the flight duration T=100 s. From this figure, there is always a UAV keeping a large transmission power. When two UAVs fly away from each other, they will increase their transmission power to improve the spectrum efficiency, e.g., from t=12 s to t=38 s. In contrast, when they serve users who are very close to each other, they will reduce their transmission power to decrease the mutual interference, e.g., from t=44 s to t=87 s. Such a joint power allocation and trajectory optimization strategy decreases the mutual interference and obtains a high average achievable data rate.

**Figure 7:**
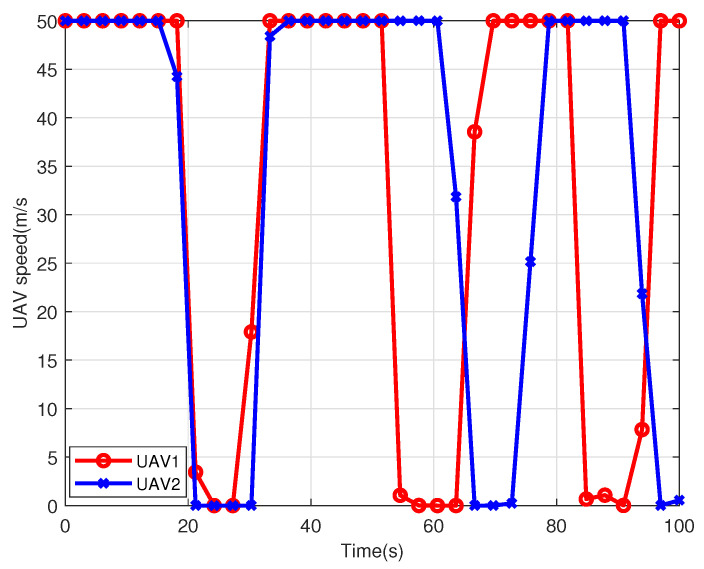
The UAV speed changes within the flight duration T=100 s in multi-UAV scenario.

Figure 7 depicts the changes of the speeds of the two UAVs within the flight duration T=100 s. From this figure, the instantaneous speed first keeps $V_{\max}=50$ m/s for a while and then drops rapidly to about 0 m/s, and holds the speed. This kind of change takes place three times in total during the flight duration. This is because each UAV sequentially serves these maritime users. It will hover over the user for a while with v=0 m/s. Then, it will fly to the next user with the maximum speed to maximize the average achievable data rate.

**Figure 8:**
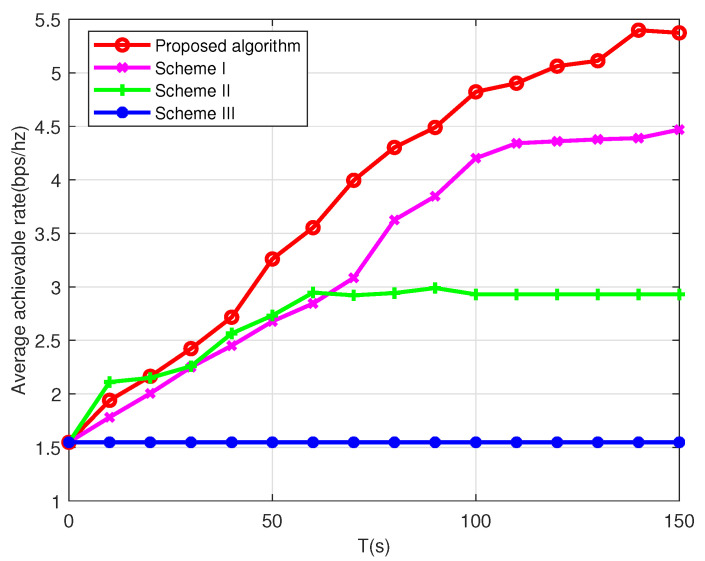
The average achievable rate for multi-UAV scenario in different schemes.

Figure 8 shows the average achievable data rate of the multi-UAV scenario under different schemes. The other three baseline schemes in [3,35] are adopted to demonstrate the effectiveness of the proposed algorithm, which are introduced as follows.

- Scheme I: The user association and UAV trajectory are jointly optimized but without transmission power allocation optimization (maximum transmission power).
- Scheme II: The user association and power allocation are jointly optimized but without trajectory optimization (the UAVs follow circular paths).
- Scheme III: The user association and power allocation are jointly optimized but with static UAVs, which are placed at the geometric center of the users.

By the performance comparison, the following observations can be made. Firstly, except for Scheme III, the max-min average achievable rates of the other three schemes increase as the flight duration *T* increases. Secondly, the performance gap between Scheme I and Scheme II increases as the flight duration *T* increases. It indicates that even without power control, the proposed trajectory optimization can achieve the throughput gain. This is because when the flight duration *T* is large, the trajectory design strategy can significantly decrease the mutual interference and achieve a large data rate. Thirdly, the performance gap between the proposed algorithm and Scheme I demonstrates that the transmission power allocation can bring additional throughput gain. The reason is that the communication interference between UAVs can be effectively reduced and the trajectory design becomes more flexible with the power control. Fourthly, by comparing the proposed algorithm and Scheme II, the proposed trajectory design also brings a significant throughput gain. Fifthly, when the UAVs are static, the max-min average achievable rate does not increase with the flight duration *T*. This is because when the UAVs are static, the communication interference between UAVs will become stable and the CSI between the UAVs and users will remain unchanged. Thus the users’ data rates will remain unchanged as well. Finally, compared with the single-UAV case in Figure 4, it can be observed that the proposed algorithm under the two-UAV case takes less time to reach the same achievable data rate. For example, to achieve the same data rate 4.5 bps/Hz, the single-UAV case needs about T=270 s, whereas the value reduces to less than T=100 s for the two-UAV case. This is because when the number of UAVs increases, the flight time of each UAV can be saved. In this situation, the UAVs have more time to hover over the users for better communication service. Therefore, it is claimed that the multi-UAV design improves the performance of the maritime communication network.

**Figure 9:**
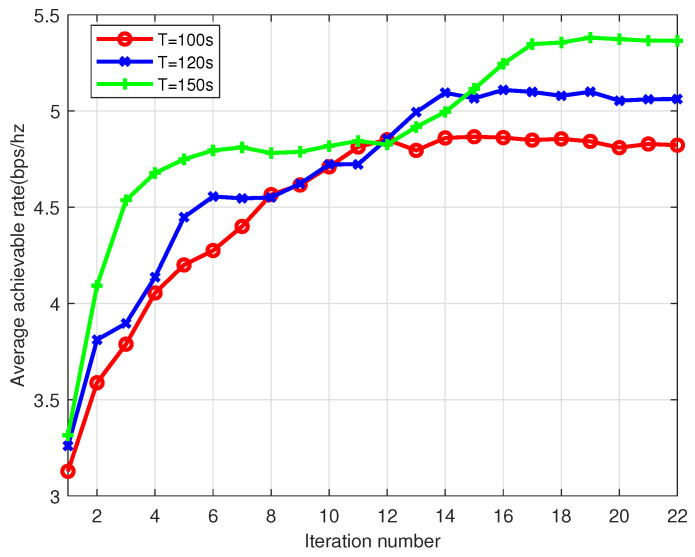
Convergence plot of Algorithm 1 for multi-UAV scenario under different flight duration *T*.

Figure 9 shows the convergence of the proposed algorithm under different flight duration *T* in the multi-UAV scenario. From this figure, the proposed algorithm can converge within several steps. Comparing the max-min average achievable rates of the different flight duration, the figure shows that the throughput increases with the flight duration. This is expected since a longer flight duration will result in the longer time to fly closer to the maritime users served by the UAVs.

## 6. Conclusions

In this paper, we investigate a multiple-UAV-enabled maritime network model. Specially, we construct the communication model by considering the effect of the maritime environment and adopt an approximation method to deal with the complexity of the two-ray channel model. The minimum average rate among all users is maximized by jointly optimizing the user association, power allocation, and UAV trajectory. To solve this non-convex problem, we divide it into three subproblems, i.e., user association optimization, power allocation optimization, and UAV trajectory optimization. By applying SCA and BCD methods, a local optimal solution with low computational complexity is achieved. Finally, extensive simulation results demonstrate the excellent performance of the proposed algorithm, as compared against several baseline schemes. Based on the results of this work, there are still many other research directions that could be further studied, such as considering the energy efficiency, mobile users, the time-varying maritime environment, and the integration of UAVs and satellites.

## Figures and Tables

**Table 1:** Notations.

Symbols	Description
K	Set of maritime users
M	Set of UAVs
*T*	Flight duration
$w_{k}$	Coordinate of user *k*
$q_{m}\left[t\right]$	Coordinate of UAV *m* in time *t*
$\delta_{t}$	Time slot
$a_{k,m}\left[n\right]$	Connection between user *k* and UAV *m* in time slot *n*
$p_{k,m}\left[n\right]$	Transmission power between user *k* and UAV *m* in time slot *n*
$h_{k,m}\left[n\right]$	Channel power gain between user *k* and UAV *m* in time slot *n*
$h_{k}$	The antenna height of user *k*
$h_{m}$	The antenna height of UAV *m*
$R_{k,m}\left[n\right]$	Data rate of user *k* served by UAV *m* in time slot *n*
A	Set of communication scheduling variables
P	Set of transmission power variables
Q	Set of UAV trajectory variables
η	Introduced slack variable
$X_{k,m}\left[n\right]$	Introduced auxiliary variable
$D_{k,m}\left[n\right]$	Introduced auxiliary variable
$z_{k,m}\left[n\right]$	Introduced auxiliary variable

**Table 2:** Simulation parameters.

Parameters	Values
Carrier frequency	2 GHz
Maximum speed	50 m/s
Maximum transmission power	1 W
Noise power	−114 dBm
Minimum collision-proof distance	100 m
Maximum iteration number	50
Threshold	$10^{-4}$

**Table 3:** Communication scheduling for two UAVs.

		UAV Index	UAV1	UAV2
	Scheduling			
User Index				
user1			0–12, 76–100 s	null
user2			13–50 s	null
user3			51–75 s	null
user4			null	0–10, 86–100 s
user5			null	11–48 s
user6			null	49–85 s

## Data Availability

Not applicable.

## Abbreviations

The following abbreviations are used in this manuscript:

UAV	Unmanned aerial vehicle
LoS	Line-of-sight
VHF	Very high frequency
TBS	Terrestrial base station
CSI	Channel state information
NOMA	Non-orthogonal multiple access
MCN	Maritime communication network
BCD	Block coordinate descent
SCA	Successive convex approximation
TDMA	Time division multiple access

## Appendix A. Proof of Lemma 1

To prove Lemma 1, we first construct the following function, as

(A1) $$g\left(x\right)=\log_{2}\left(ax+b\right),$$

where the constant a≥0,b≥0. By taking the second derivative of this function, we have

(A2) $$g{}^{\prime \prime }\left(x\right)=\frac{-a^{2}}{{\left(ax+b\right)}^{2}\ln2}.$$

It can be observed that $g{}^{\prime \prime }\left(x\right)\leq 0$. Therefore, g(x) is a concave function. The form of ${\hat{R}}_{k,m}\left[n\right]$ is similar to g(x), so ${\hat{R}}_{k,m}\left[n\right]$ is a concave function with respect to variable $p_{k,j}\left[n\right]$. According to [36], any concave function is globally upper-bounded by its first-order Taylor expansion at any given point. With this theorem, we can first obtain the derivative of ${\hat{R}}_{k,m}\left[n\right]$, as

(A3) $$\begin{array}{cc} \; & {\left.\frac{\partial {\hat{R}}_{k,m}\left[n\right]}{\partial p_{k,j}\left[n\right]}\right|}_{p_{k,j}\left[n\right]=p_{k,j}^{l}\left[n\right]}\\ = & \frac{\partial \left[\log_{2}\left(\sum_{j=1,j\neq m}^{M}p_{k,j}^{l}\left[n\right]h_{k,j}\left[n\right]+\sigma^{2}\right)\right]}{\partial p_{k,j}^{l}\left[n\right]}\\ = & \frac{h_{k,j}\left[n\right]}{\sum_{i=1,i\neq m}^{M}p_{k,i}^{l}\left[n\right]h_{k,i}\left[n\right]+\sigma^{2}}\\ ≜ & B_{k,j}^{l}\left[n\right]. \end{array}$$

Then, the upper bound of ${\hat{R}}_{k,m}\left[n\right]$ can be obtained, as

(A4) $$\begin{array}{cc} {\hat{R}}_{k,m}\left[n\right] & \leq \sum_{j=1,j\neq m}^{M}B_{k,j}^{l}\left[n\right]\left(p_{k,j}\left[n\right]-p_{k,j}^{l}\left[n\right]\right)\\ \; & +\log_{2}\left(\sum_{j=1,j\neq m}^{M}p_{k,j}^{l}\left[n\right]h_{k,j}\left[n\right]+\sigma^{2}\right). \end{array}$$

It is obvious that ${\hat{R}}_{k,m}\left[n\right]$ is linear with respect to variable $p_{k,j}\left[n\right]$ since the coefficient $B_{k,j}^{l}\left[n\right]$ is a positive constant.

## Appendix B. Proof of Lemma 2

We all know that $f\left(x\right)=e^{-x}$ is a convex function, since its second derivative is greater than or equal to zero. Similarly, we define $f\left(z_{k,j}\left[n\right]\right)=\log_{2}\left(\sum_{j=1,j\neq m}^{M}e^{z_{k,j}\left[n\right]}+\sigma^{2}\right)$. If we take the second partial derivative of it, we can obtain

(A5) $$f{}^{\prime \prime }\left(z_{k,j}\left[n\right]\right)=\frac{\sum_{i=1,i\neq j,i\neq m}^{M}e^{z_{k,i}\left[n\right]}+\sigma^{2}}{{\left(\sum_{j=1,j\neq m}^{M}e^{z_{k,j}\left[n\right]}+\sigma^{2}\right)}^{2}}.$$

The numerator and denominator of $f{}^{\prime \prime }\left(z_{k,j}\left[n\right]\right)$ are both greater than 0, so $f{}^{\prime \prime }\left(z_{k,j}\left[n\right]\right)\geq 0$. Therefore, we examine the Hessian matrix of $f(z_{k,j}\left[n\right])$ and conclude that $f(z_{k,j}\left[n\right])$ is convex.

## Appendix C. Proof of Lemma 3

To prove Lemma 3, we first construct the following function, as

(A6) $$f\left(x\right)=\log_{2}\left(a+\frac{b}{x^{2}}\right),$$

where the constant b≥0. By taking the second derivative of this function, we have

(A7) $$f{}^{\prime \prime }\left(x\right)=\frac{6abx^{2}+2b^{2}}{{\left(ax^{3}+bx\right)}^{2}\ln2}.$$

It can be observed that $f{}^{\prime \prime }\left(x\right)\geq 0$. Therefore, f(x) is convex. According to [36], any convex function is globally lower-bounded by its first-order Taylor expansion at any given point. For given point $x_{0}$, we have

(A8) $$f\left(x\right)\geq f\left(x_{0}\right)+f{}^{\prime }\left(x_{0}\right)\left(x-x_{0}\right),$$

where

(A9) $$f{}^{\prime }\left(x_{0}\right)=\frac{-2b}{\left(ax_{0}^{3}+bx_{0}\right)\ln2},$$

is the derivative of f(x) at point $x_{0}$. Thus, the following equality holds, as

(A10) $$\log_{2}\left(a+\frac{b}{x^{2}}\right)\geq \log_{2}\left(a+\frac{b}{x_{0}^{2}}\right)+\frac{-2b}{\left(ax_{0}^{3}+bx_{0}\right)\ln2}\left(x-x_{0}\right).$$

Next, similar to Appendix A, by taking the derivative of ${\overset{ˇ}{R}}_{k,m}\left[n\right]$, we have

(A11) $$\begin{array}{cc} \; & {\left.\frac{\partial {\overset{ˇ}{R}}_{k,m}\left[n\right]}{\partial \left({∥q_{j}\left[n\right]-w_{k}∥}^{2}\right)}\right|}_{{∥q_{j}\left[n\right]-w_{k}∥}^{2}={∥q_{j}^{l}\left[n\right]-w_{k}∥}^{2}}\\ = & \frac{\partial \left[\log_{2}\left(\sum_{i=1}^{M}(\frac{p_{k,i}\left[n\right]{\left(h_{k}h_{m}\right)}^{2}}{{\left({∥q_{i}^{l}\left[n\right]-w_{k}∥}^{2}\right)}^{2}}+\sigma^{2}\right)\right]}{\partial \left({∥q_{j}^{l}\left[n\right]-w_{k}∥}^{2}\right)}\cdot \frac{\partial \frac{p_{k,j}\left[n\right]{\left(h_{k}h_{m}\right)}^{2}}{{\left({∥q_{j}\left[n\right]-w_{k}∥}^{2}\right)}^{2}}}{\partial \left({∥q_{j}^{l}\left[n\right]-w_{k}∥}^{2}\right)}\\ = & \frac{\log_{2}\left(e\right)}{\sum_{i=1}^{M}\left(\frac{p_{k,i}\left[n\right]{\left(h_{k}h_{m}\right)}^{2}}{{\left({∥q_{i}^{l}\left[n\right]-w_{k}∥}^{2}\right)}^{2}}+\sigma^{2}\right)}\cdot \frac{-2p_{k,j}\left[n\right]{\left(h_{k}h_{m}\right)}^{2}}{{\left({∥q_{j}^{l}\left[n\right]-w_{k}∥}^{2}\right)}^{3}}\\ = & \frac{-2\log_{2}\left(e\right)p_{k,j}\left[n\right]{\left(h_{k}h_{m}\right)}^{2}}{{\left({∥q_{j}^{l}\left[n\right]-w_{k}∥}^{2}\right)}^{3}\sum_{i=1}^{M}\left(\frac{p_{k,i}\left[n\right]{\left(h_{k}h_{m}\right)}^{2}}{{\left({∥q_{i}^{l}\left[n\right]-w_{k}∥}^{2}\right)}^{2}}+\sigma^{2}\right)}\\ ≜ & -I_{k,j}^{l}\left[n\right]. \end{array}$$

Let $a=\sigma^{2}$, $b=p_{k,i}\left[n\right]{\left(h_{k}h_{m}\right)}^{2}$ and $x_{0}={∥q_{j}^{l}\left[n\right]-w_{k}∥}^{2}$. Then, the lower bound of ${\overset{ˇ}{R}}_{k,m}\left[n\right]$ can be obtained as

(A12) $$\begin{array}{cc} {\overset{ˇ}{R}}_{k,m}\left[n\right] & \geq \log_{2}\left(\sum_{i=1}^{M}(\frac{p_{k,i}\left[n\right]{\left(h_{k}h_{m}\right)}^{2}}{{\left({∥q_{i}^{l}\left[n\right]-w_{k}∥}^{2}\right)}^{2}}+\sigma^{2}\right)\\ \; & +\sum_{j=1}^{M}-I_{k,j}^{l}\left[n\right]\left({∥q_{j}\left[n\right]-w_{k}∥}^{2}-{∥q_{j}^{l}\left[n\right]-w_{k}∥}^{2}\right). \end{array}$$

It is obvious that ${\overset{ˇ}{R}}_{k,m}\left[n\right]$ is linear to variable ${∥q_{j}\left[n\right]-w_{k}∥}^{2}$, since the coefficient $I_{k,j}^{l}\left[n\right]$ is a positive constant.

## References

[1] Wei T., Feng W., Chen Y., Wang C.-X., Ge N., Lu J. (2021). Hybrid satellite-terrestrial communication networks for the maritime internet of things: Key technologies, opportunities, and challenges. IEEE Internet Things J..

[2] Li H., Yu C., Zhang C., Jiao H., Lin B., He R. Maritime multi-relay communications based on UAV trajectory adjustment and dual Q-learning. Proceedings of the 2021 International Conference on Security, Pattern Analysis, and Cybernetics.

[3] Zeng C., Wang J.B., Ding C., Zhang H., Lin M., Cheng J. (2021). Joint optimization of trajectory and communication resource allocation for unmanned surface vehicle enabled maritime wireless networks. IEEE Trans. Commun..

[4] Li B., Fei Z., Zhang Y. (2019). UAV communications for 5G and beyond: Recent advances and future trends. IEEE Internet Things J..

[5] Li X., Feng W., Wang J., Chen Y., Ge N., Wang C.X. (2020). Enabling 5G on the ocean: A hybrid satellite-UAV-terrestrial network solution. IEEE Wirel. Commun..

[6] Tang R., Feng W., Chen Y., Ge N. (2021). NOMA-based UAV communications for maritime coverage enhancement. China Commun..

[7] Hou Q., Cai Y., Hu Q., Lee M., Yu G. (2022). Joint resource allocation and trajectory design for multi-UAV systems with moving Users: Pointer network and unfolding. IEEE Trans. Wirel. Commun..

[8] Naqvi S.A.R., Hassan S.A., Pervaiz H., Ni Q. (2018). Drone-aided communication as a key enabler for 5G and resilient public safety networks. IEEE Commun. Mag..

[9] Zeng Y., Lyu J., Zhang R. (2019). Cellular-connected UAV: Potential, challenges, and promising technologies. IEEE Wirel. Commun..

[10] Wang J., Zhou H., Li Y., Sun Q., Wu Y., Jin S., Quek T.Q.S., Xu C. (2018). Wireless channel models for maritime communications. IEEE Access.

[11] Balkees P.A.S., Sasidhar K., Rao S. A survey based analysis of propagation models over the sea. Proceedings of the 6th International Conference on Advances in Computing, Communications and Informatics.

[12] Wei T., Feng W., Wang J., Ge N., Lu J. (2019). Exploiting the shipping lane information for energy-efficient maritime communications. IEEE Trans. Veh. Technol..

[13] Liu C., Feng W., Wei T., Ge N. (2018). Fairness-oriented hybrid precoding for massive MIMO maritime downlink systems with large-scale CSIT. China Commun..

[14] Jo S.W., Shim W.S. (2019). LTE-maritime: High-speed maritime wireless communication based on LTE technology. IEEE Access.

[15] Su N., Wang J.B., Zeng C., Zhang H., Lin M., Li G.Y. (2022). Unmanned surface vehicle aided maritime data collection using deep reinforcement learning. IEEE Internet Things J..

[16] Li X., Feng W., Chen Y., Wang C.X., Ge N. (2020). Maritime coverage enhancement using UAVs coordinated with hybrid satellite-terrestrial networks. IEEE Trans. Commun..

[17] Yang T., Jiang Z., Sun R., Cheng N., Feng H. (2020). Maritime search and rescue based on group mobile computing for unmanned aerial vehicles and unmanned surface vehicles. IEEE Trans. Ind. Informat..

[18] Mozaffari M., Saad W., Bennis M., Debbah M. (2016). Efficient deployment of multiple unmanned aerial vehicles for optimal wireless coverage. IEEE Commun. Lett..

[19] Dai H., Bian H., Li C., Wang B. (2020). UAV-aided wireless communication design with energy constraint in space-air-ground integrated green IoT networks. IEEE Access.

[20] Kumar K., Kumar S., Kaiwartya O., Sikandar A., Kharel R., Mauri J.L. (2020). Internet of unmanned aerial vehicles: QoS provisioning in aerial ad-hoc networks. Sensors.

[21] Chiaraviglio L., D’Andreagiovanni F., Liu W., Gutierrez J.A., Blefari-Melazzi N., Choo K.K.R., Alouini M.S. (2021). Multi-area throughput and energy optimization of UAV-aided cellular networks powered by solar panels and grid. IEEE Trans. Mobile Comput..

[22] Zeng Y., Zhang R. (2017). Energy-efficient UAV communication with trajectory optimization. IEEE Trans. Wirel. Commun..

[23] Wang Y., Feng W., Wang J., Quek T.Q.S. (2021). Hybrid satellite-UAV-terrestrial networks for 6G ubiquitous coverage: A maritime communications perspective. IEEE J. Sel. Areas Commun..

[24] Feng W., Wang Y., Ge N., Lu J., Zhang J. (2013). Virtual MIMO in multi-cell distributed antenna systems: Coordinated transmissions with large-scale CSIT. IEEE J. Sel. Areas Commun..

[25] Feng W., Wang Y., Lin D., Ge N., Lu J., Li S. (2017). When mmWave communications meet network densification: A scalable interference coordination perspective. IEEE J. Sel. Areas Commun..

[26] Jha S.K., Prakash S., Rathore R.S., Mahmud M., Kaiwartya O., Lloret J. (2022). Quality-of-service-centric design and analysis of unmanned aerial vehicles. Sensors.

[27] Yang K., Roste T., Bekkadal F., Ekman T. Channel characterization including path loss and Doppler effects with sea reflections for mobile radio propagation over sea at 2 GHz. Proceedings of the 2nd International Conference on Wireless Communications and Signal Processing.

[28] Mengali U., D’Andrea A.N. (2013). Synchronization Techniques for Digital Receivers.

[29] Gueye S., Michel S., Yassine A. A 0-1 linear programming formulation for the Berth Assignment Problem. Proceedings of the 4th International Conference on Logistics.

[30] Todd M.J. (1992). A low complexity interior-point algorithm for linear programming. SIAM J. Optim..

[31] Zhong C., Yao J., Xu J. (2019). Secure UAV communication with cooperative jamming and trajectory control. IEEE Commun. Lett..

[32] Li Y., Zhang R., Zhang J., Gao S., Yang L. (2019). Cooperative jamming for secure UAV communications with partial eavesdropper information. IEEE Access.

[33] Lee H., Eom S., Park J., Lee I. (2018). UAV-aided secure communications with cooperative jamming. IEEE Trans. Veh. Technol..

[34] Larsson A., Piotrowski A., Giles T., Smart D. Near-earth RF propagation—Path loss and variation with weather. Proceedings of the 2nd International Conference on Radar.

[35] Wu Q., Zeng Y., Zhang R. (2018). Joint trajectory and communication design for multi-UAV enabled wireless networks. IEEE Trans. Wirel. Commun..

[36] Boyd S., Vandenberghe L., Faybusovich L. (2006). Convex optimization. IEEE Trans. Autom. Control.

